# Remote cerebellar hemorrhage following repeated lumbar punctures

**DOI:** 10.1186/s12883-023-03276-6

**Published:** 2023-06-08

**Authors:** Hai-Yang Wang, Zerui Hu, Jinming Han, Dongsen Wang, Qingjian Wu

**Affiliations:** 1 Department of Neurology, Jining No.1 People’s Hospital, Jining, 272000 Shandong Province China; 2 Department of Psychiatry, School of Mental Health, Jining Medical University, Jining, 272000 China; 3 Department of Neurology, Xuanwu Hospital, Capital Medical University, Beijing, 100053 China; 4 Clinical Medical College of Jining Medical University, Jining, Shandong Province, 272067 China; 5 Department of Emergency, Jining No.1 People’s Hospital, No. 6, Jiankang Road, Jining, 272011 Shandong Province China

**Keywords:** Remote cerebellar hemorrhage, Repeated lumbar punctures, Meningoencephalitis, Zebra sign, MRI

## Abstract

**Background:**

Remote cerebellar hemorrhage (RCH) is a rare complication in neurosurgery. No case of RCH secondary to repeated lumbar punctures (LPs) has been previously reported.

**Case presentation:**

A 49-year-old man presented with impaired consciousness following persistent fever. Cerebrospinal fluid examination showed high opening pressure, elevated white blood cells, increased protein level, and decreased glucose level, resulting in a diagnosis of bacterial meningoencephalitis. Treatment with repeated LPs and intrathecal injection of ceftriaxone resulted in an improvement in neurological symptoms. However, on day 31 of treatment, brain magnetic resonance image (MRI) showed streaky bleeding in bilateral cerebellum (zebra sign), leading to a diagnosis of RCH. Close observation and repeated brain MRI imaging without specific treatments led to the absorption of bilateral cerebellar hemorrhage, and the patient was discharged with improved neurological symptoms. Repeated brain MRI scans one month after discharge showed that bilateral cerebellar hemorrhage had improved, and had disappeared one year after discharge.

**Conclusion:**

We reported a rare occurrence of LPs-induced RCH presenting as isolated bilateral inferior cerebellar hemorrhage. Clinicians should be vigilant of the risk factors for RCH, closely monitoring patients' clinical symptoms and neuroimaging findings to determine the need for specialized treatment. Furthermore, this case highlights the importance of ensuring the safety of LPs and managing any potential complications appropriately.

## Background

Remote cerebellar hemorrhage (RCH) is an unusual complication of supratentorial or spinal operations, with an incidence being 0.08%-0.6% [1, 2]. It is well-documented that head rotation, cerebrospinal drainage, and the use of antiplatelet or anticoagulation drugs can cause RCH [1–3]. Other conditions associated with RCH include postoperative unruptured aneurysm and spinal surgery [4, 5]. To our knowledge, RCH following LPs has not been reported in the literature. Herein, we reported a rare case of RCH secondary to repeated LPs (due to meningoencephalitis) with streaky bleeding in bilateral cerebellum. Long-term clinical and neuroimaging follow-ups were performed to demonstrate the dynamic evolution of this case.

## Case presentation

A 49-year-old male patient was found to be unconscious following five days of persistent fever. His family brought him to the emergency room due to altered mental status, and he was subsequently transferred to our Intensive Care Unit. The patient had no past medical history, no history of head, spinal, or hip trauma, no record of medication use, and no family history of medical conditions. Neurological examinations revealed that the patient had altered consciousness, respiratory distress, a pupil diameter of 3.0 mm, sluggish response to light reflex, eye movement disorder associated with brainstem dysfunction, muscle strength of grade 3 in the right limb, grade 2 in the left limb, positive pathological signs on the right side, and neck stiffness. The patient has a Glasgow Coma Scale score of 5, reflecting severe neurological impairment. Cranial CT examination on the day of admission revealed no significant abnormalities. Laboratory tests revealed leucocytosis (white blood cells, 20.58 × 10^9^/L), thrombocytopenia (23 × 10^9^/L), elevated serum procalcitonin (21.37 ng/mL), increased erythrocyte sedimentation rate (25 mm/h), decreased serum albumin (27.4 g/L), elevated creatine kinase (509 U/L), hyperglycemia (17.6 mmol/L), and elevated D-dimer (3935 ng/ml). Blood culture, tumor markers, homocysteine, coagulation factors, blood creatinine, and liver function tests were unremarkable. The significant thrombocytopenia to be a consequence of severe infection in its early stage [6], as the patient had no symptoms or history of thrombocytopenia, nor had taken any anticoagulant or antiplatelet medications. LP was not performed immediately due to the severe thrombocytopenia, and the platelets (3 units) were transfused to prevent bleeding. After treatment, the patient’s thrombocyte count rose to 120 × 10^9^/L. Following the absence of any contraindications to LP, the procedure was performed to obtain Cerebrospinal fluid (CSF) for diagnostic purposes. A 20-gauge spinal needle was used for the procedure, and the patient was instructed to lie flat on their back without a pillow for 4 to 6 h after the procedure. CSF examination showed a yellow fluid with a high opening pressure of 280 mmH_2_O, elevated white blood cells (9600 × 10^6^/L) with 90% being neutrophils, elevated protein level (135 mg/dl) and decreased glucose level (1.10 mmol/L). Anti-body of virus and tuberculosis-DNA was not detected and pandy test is positive. CSF levels of IgA, IgG, and IgM were 26.8 mg/L, > 117 mg/L, and 3.29 mg/L, respectively. Viral, fungal, and bacterial PCR, cultures, and cytology were negative and no malignant cells were found in the CSF. A diagnosis of bacterial meningoencephalitis was finally established.

**Fig. 1:**
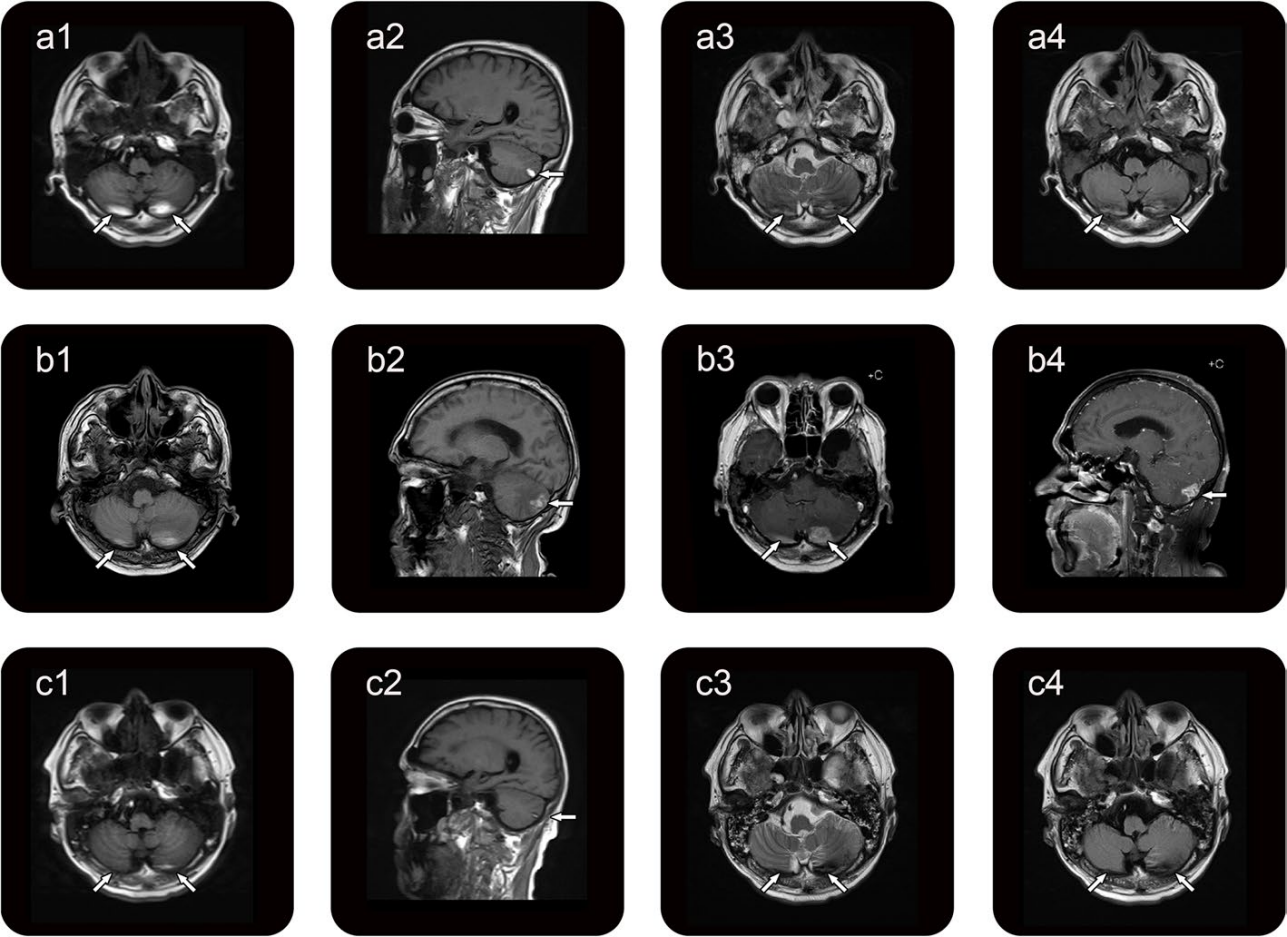
The brain MRI scans during hospitalization. On day 31, brain MRI showed streaky hyperintensity of T1WI (a1, a2; arrows) and streaky alternate hyperintensity and hypointensity of the T2WI (a3, arrows) and T2 FLAIR (a4, arrows) in bilateral cerebellum. On day 42, brain MRI shows T1WI punctate and lamellar enhancement (b3, b4; arrows) with streaky T1WI hyperintensity (b1, b2; arrows) in bilateral cerebellum. On day 57, brain MRI demonstrated the absorption of bilateral cerebellar hemorrhage compared to previous ones with a decreased signal intensity on T1WI (c1, c2; arrows), T2WI (c3, arrows) and T2 FLAIR (c4, arrows)

An early-stage rapid brain CT scan was conducted to evaluate for organic brain lesions in a critically ill patient with impaired consciousness. No hemorrhage was found on the scan. We performed ten diagnostic and therapeutic LPs with intrathecal injection of ceftriaxone (50 mg once a day) and the patient's neurological symptoms improved, such as consciousness, speech and movement. In the subsequent LPs, the CSF pressure was found to range between 130 mmH_2_O and 320 mmH_2_O, showing a declining trend. The pressure was measured at 140 mmH_2_O in the latest test. On the 31st day of hospitalization, following the completion of four lumbar punctures, the patient exhibited some improvement in symptoms. To ascertain the presence of intracranial pathology, a brain MRI examination was conducted. The results revealed bilateral cerebellar hemorrhage, appearing as the ‘zebra sign’ with streaky hyperintensity on T1-weighted images (T1WI) (Fig. 1, a1 and a2) and streaky alternate hyperintensity and hypointensity on the T2-weighted image (T2WI) (Fig. 1, a3) and the T2 fluid attenuated inversion recovery (T2 FLAIR) image (Fig. 1, a4) in the bilateral cerebellum. Notably, the patient did not have any cerebellar symptoms. Cerebral hemorrhage was not observed on brain CT during early treatment and was subsequently detected on brain MRI after LPs. Based on a history of repeated LPs and brain MRI scans, a diagnosis of RCH was considered by ruling out other potential possibilities. As RCH is typically a self-limiting disease [2], we closely observed the patient and conducted repeated brain MRI imaging without any specific treatments.

On day 42, bilateral cerebellar hemorrhage with punctate and/or lamellar enhancement and the streaky hyperintensity on T1WI (Fig. 1, b1-b4) was evident in bilateral cerebellum. On day 57, a repeated brain MRI showed the absorption of bilateral cerebellar hemorrhage compared to previous scans, with decreased signal intensity on T1WI (Fig. 1, c1 and c2), T2WI (Fig. 1, c3) and T2 FLAIR (Fig. 1, c4). Two months later, the patient was discharged with significantly improved neurological symptoms.

**Fig. 2:**
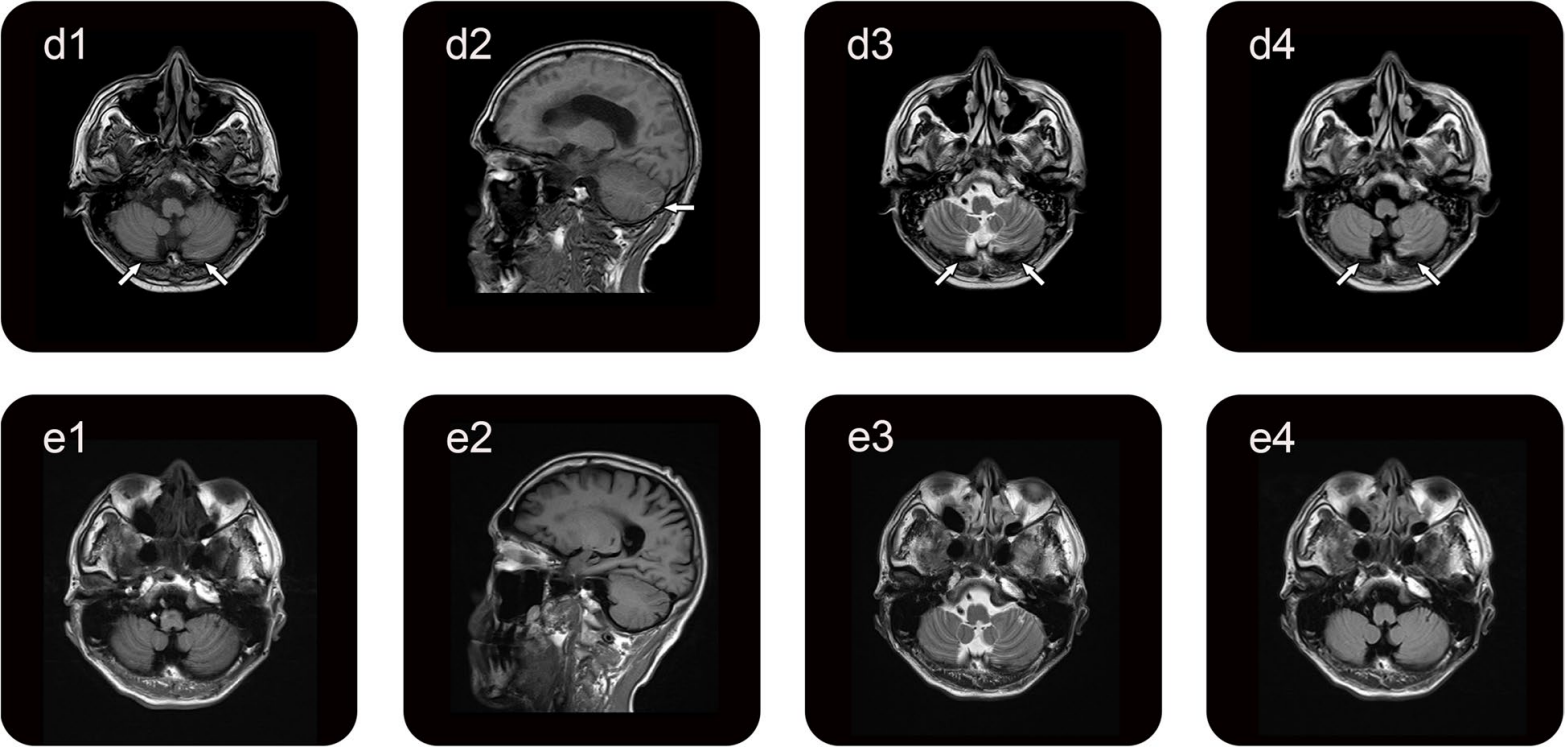
The follow-up brain MRI scans after discharge. Brain MRI, 1 month later, showed a little streaky T1WI hyperintensity (d1, d2; arrows) with T2WI and T2 FLAIR hypointensity (d3, d4; arrows). A follow-up brain MRI, 1 year later, showed no abnormal findings (e1-e4)

During the follow-up periods, the patient reported significant improvement of clinical symptoms. A repeated brain MRI scan one month after discharge showed that bilateral cerebellar hemorrhage had improved (Fig. 2, d1-d4). Fortunately, the cerebellar hemorrhage had disappeared one year after discharge (Fig. 2, e1-e4).

## Discussion

We report a rare condition of RCH, probably caused by multiple LPs, which presented with a typical ‘zebra sign’ of streaky hyperintensities on TIWI in the bilateral cerebellar hemispheres.

RCH is a rare complication of neurosurgery characterized by spontaneous posterior fossa hemorrhage with a streaky bleeding pattern caused by blood spreading parallel to the cerebellar foliae. The typical ‘zebra sign’ can be noted in approximately 65% RCH patients [2]. The main clinical symptoms of RCH include headache, ataxia and a decreased level of consciousness [1, 2]. In our case, no obvious neurological deficits due to RCH were observed. Instead, no obvious neurological deficits due to RCH were observed. Instead, cerebellar hemorrhage was only detected on brain MRI after lumbar puncture, and not on brain CT during early treatment, which confirmed the diagnosis. The absence of significant neurological alterations in this patient may be due to the bleeding being inferiorly located, with no hemorrhage or edema compressing the brainstem or hydrocephalus. Therefore, clinicians should be aware of the possibility of cerebellar hemorrhage in patients with repeated LPs, even if they do not present any obvious symptoms.

The exact pathophysiology of RCH is not yet fully understood, but several mechanisms have been proposed based on clinical observations and imaging studies. One proposed mechanism is that RCH is caused by changes in CSF dynamics following neurosurgical procedures. Excessive CSF drainage or sudden changes in intracranial pressure (ICP) may lead to a shift in the pressure gradient between the intracranial and spinal compartments, resulting in the rupture of small vessels in the cerebellum and subsequent hemorrhage [1, 2]. The loss of CSF is considered to be a dominant contributor to RCH [7–9]. Another possible mechanism is that RCH is caused by changes in blood flow and pressure within the brain. It has been proposed that the sudden release of pressure following decompressive surgery or trauma may lead to a rebound increase in cerebral blood flow [10], which can cause rupture of fragile vessels in the cerebellum. Additionally, some studies have suggested that RCH may be caused by a combination of both CSF and blood flow-related mechanisms [9]. In our case, multiple LPs may have resulted in a significant loss of CSF and decreased ICP, leading to cerebellar subsidence. We propose that stretching of the cerebellar veins due to subsidence may contribute to cerebellar hemorrhage. Thus, a sudden drop in intracranial pressure caused by the loss of CSF from multiple LPs and the displacement of brain tissue and blood vessels may be the mechanisms of RCH.

The loss of CSF is primarily associated with the development of CSF leakage development after neurosurgical procedures, which can result in RCH [2]. High ICP and elevated CSF pressure are significant risk factors for CSF leakage following neurosurgical procedures [11–13] and are associated with prolonged hospitalization and postoperative complications [14]. In a study by Teachey et al., continuous monitoring and management of ICP through the use of an external ventricular drain significantly improved the success rate following the repair of CSF leakage [15]. CSF drainage and surgical decompression are commonly used methods for reducing ICP and preventing CSF leakage [16]. These studies suggest that controlling high ICP and CSF pressure is a crucial aspect of managing CSF leakage after neurosurgical procedures. Therefore, high ICP and CSF pressure may have contributed to the CSF leakage, leading to RCH in our case. Additionally, the patient's infection may have caused abnormal coagulation and reduced vascular bleeding threshold [17], which could have contributed to cerebellar hemorrhage, although this cannot be fully excluded.

Neuroimaging was not immediately performed after LPs, and the discovery of RCH was incidental during a later routine MRI examination, which may have led to a delayed diagnosis. Additionally, the absence of obvious neurological symptoms made the identification of RCH more difficult. Fortunately, no serious adverse events occurred due to close observation of this patient.

In conclusion, this case demonstrates a rare occurrence of LPs-induced remote cerebellar hemorrhage (RCH) presenting as isolated bilateral inferior cerebellar hemorrhages. Clinicians should be vigilant of the risk factors for RCH, closely monitoring patients' clinical symptoms and neuroimaging findings to determine the need for specialized treatment. It is also important to conduct neuroimaging following LPs to detect possible RCH and avoid delayed diagnosis. Furthermore, this case highlights the importance of ensuring the safety of LPs, releasing CSF slowly, and managing any potential complications appropriately to improve patient outcomes.

## Data Availability

The supporting data for the findings of this case are accessible upon reasonable request from the corresponding author, but not publicly available due to ethical or privacy constraints.

## Abbreviations

RCH: Remote cerebellar hemorrhage
LPs: Lumbar punctures
MRI: Brain magnetic resonance image
CSF: Cerebrospinal fluid
T1WI: T1-weighted images
T2WI: T2-weighted image
T2 FLAIR: T2 fluid attenuated inversion recovery
